# A Case of Semicircular Lipoatrophy

**DOI:** 10.7759/cureus.114097

**Published:** 2026-08-06

**Authors:** Rita Diz, Elisabete Mendes, Andreia Diegues, Rita Pera, Helena Maurício

**Affiliations:** 1 Department of Internal Medicine, Unidade Local de Saúde do Nordeste, Bragança, PRT; 2 Department of Internal Medicine, Unidade Local de Saúde do Alto Alentejo, Portalegre, PRT

**Keywords:** lipoatrophia, lipoatrophy, microtrauma, semicircular, workplace injuries

## Abstract

We present a case of a 34-year-old woman with semicircular lipoatrophy, characterized by linear skin depressions in a semicircular band-like atrophy on the anterolateral aspects of the thighs. It is still debated whether this type of occurrence is rare or simply underreported because it does not cause overt suffering to the patient, but its estimated incidence has been increasing. The most plausible explanation appears to be repeated external microtrauma, as avoiding its repetition results in complete remission of semicircular lipoatrophy in most cases.

## Introduction

Semicircular lipoatrophy (SL) is a rare condition characterized by semicircular, band-like horizontal depressions of the skin, typically affecting the anterior and lateral aspects of the thighs [1]. It represents a benign and usually reversible form of localized subcutaneous tissue atrophy, with most reported cases occurring in women [1]. Although the exact pathogenesis remains unclear, repetitive external mechanical pressure is considered the most widely accepted contributing factor, particularly in occupational settings; however, the triggering factors may not always be readily identified [2].

Despite traditionally being considered an uncommon disorder, the number of reported SL cases has increased substantially since the mid-2000s, mainly due to the recognition of workplace-related outbreaks in modern office environments and increased clinical awareness, rather than necessarily reflecting a true increase in incidence [3-5].

This case highlights the diagnostic challenges of SL, especially when the association with repetitive occupational microtrauma is not initially recognized. Awareness of this condition and a detailed assessment of workplace exposure are essential to ensure timely diagnosis and to allow the implementation of appropriate preventive measures.

## Case presentation

In this report, we describe a case of a 34-year-old woman, a worker at a store, who presented to the emergency department (ED) with bilateral linear skin depressions involving the anterior and lateral aspects of both thighs. She had noticed the lesions several days earlier and subsequently sought medical evaluation at a health center, where a muscle disorder was suspected and topical nonsteroidal anti-inflammatory drugs (NSAIDs) were prescribed. As there was no clinical improvement, she returned to the ED.

The patient denied lower limb pain, inflammatory skin changes, paresthesia, systemic symptoms, or any relationship between the lesions and menstruation. She also denied wearing tight clothing or previous trauma. Her medical and medication history was unremarkable.

**Figure 1:**
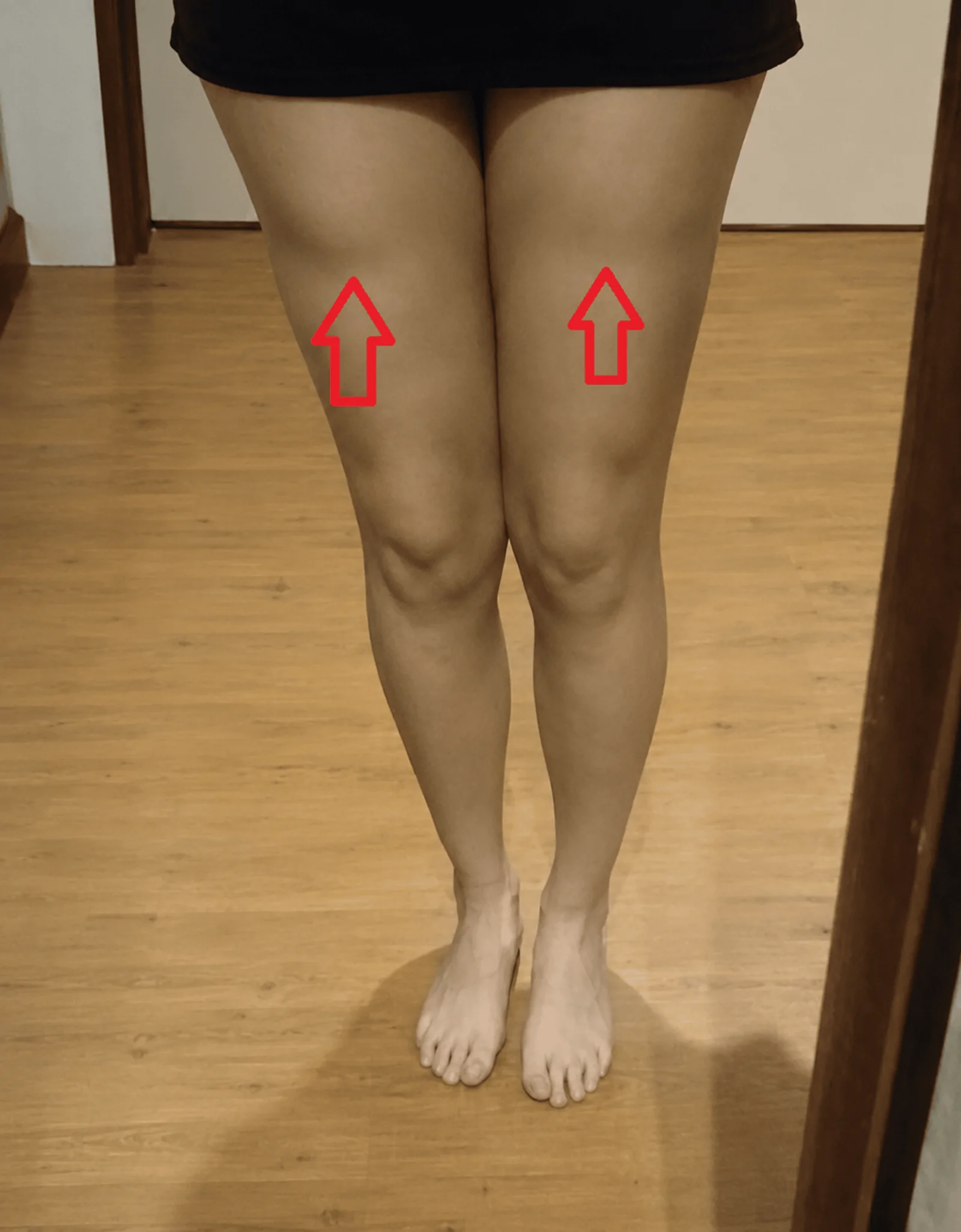
Bilateral, symmetrical linear depressions (red arrows) on the anterolateral mid-thighs, forming a characteristic semicircular band-like pattern of localized lipoatrophy. The overlying skin appears normal, with no erythema or induration.

**Figure 2:**
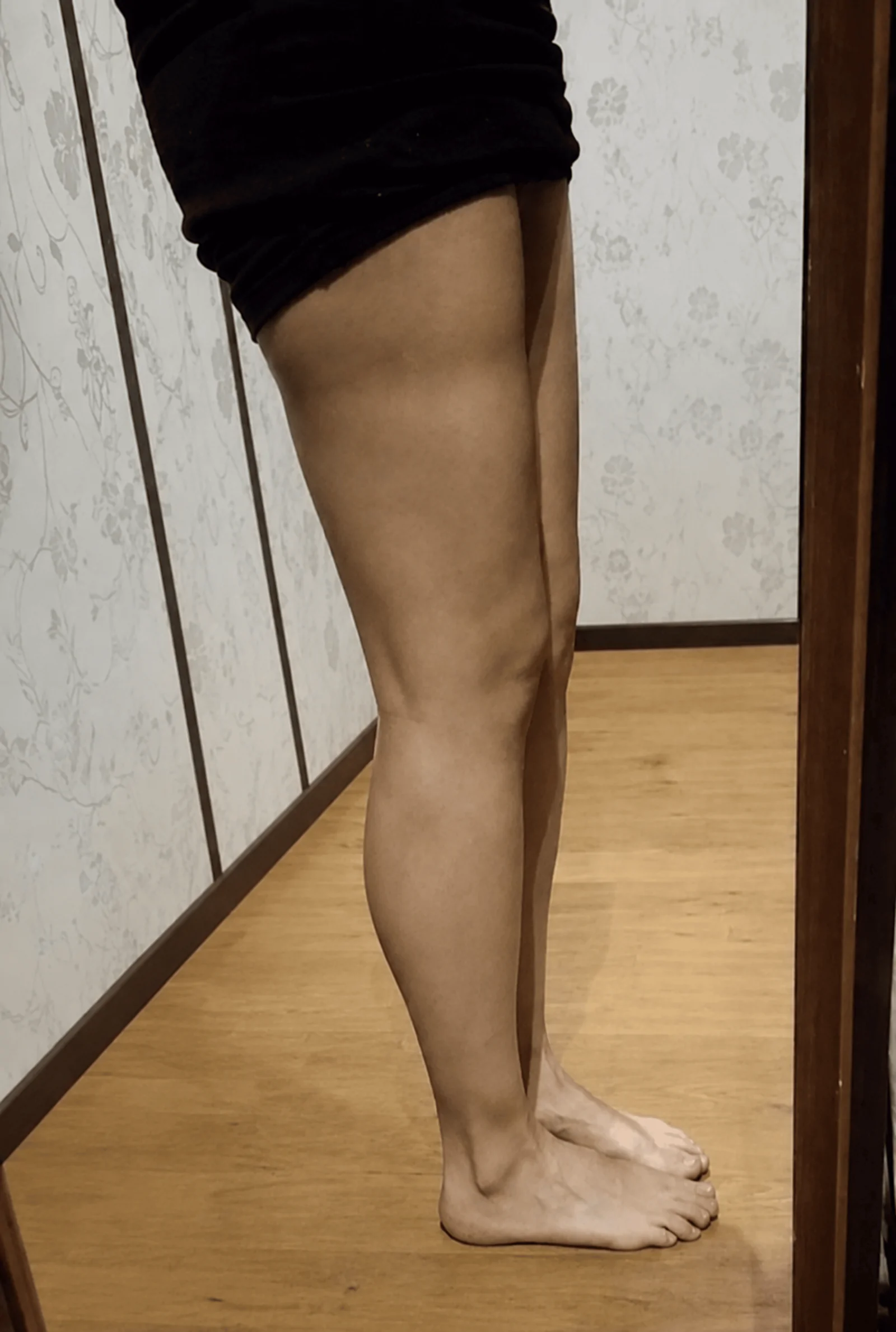
Bilateral linear skin depressions, with the right-sided lesion more clearly visible, forming a characteristic semicircular band-like pattern of localized lipoatrophy.

Physical examination revealed bilateral linear depressions with a semicircular band-like distribution on the anterolateral aspects of the thighs, with normal overlying skin (Figures 1, 2). Laboratory evaluation, including complete blood count and autoimmune serology (antinuclear antibody and extractable nuclear antigen antibodies), as well as arterial and venous Doppler ultrasonography of both lower limbs, showed no abnormalities. Soft tissue ultrasonography demonstrated reduced subcutaneous tissue in the middle third of both thighs, with preservation of the underlying fascia and muscle structures.

These findings were consistent with localized loss of subcutaneous adipose tissue and supported the diagnosis of semicircular lipoatrophy (SL) in the appropriate clinical context. No laboratory or vascular abnormalities suggestive of systemic, autoimmune, or vascular disease were identified. Additional imaging, including magnetic resonance imaging, was not considered necessary, as the clinical examination and ultrasound findings showed no evidence of muscle, fascial, or inflammatory involvement and were unlikely to provide additional diagnostic information.

Histopathological confirmation was not obtained because the patient declined biopsy. Nevertheless, the diagnosis was established based on the characteristic clinical appearance, bilateral distribution, compatible ultrasonographic findings, absence of inflammatory signs or systemic manifestations, and subsequent identification of a repetitive mechanical trigger. The lack of pain, erythema, induration, or fascial involvement made alternative diagnoses, such as morphea or localized panniculitis, less likely.

**Figure 3:**
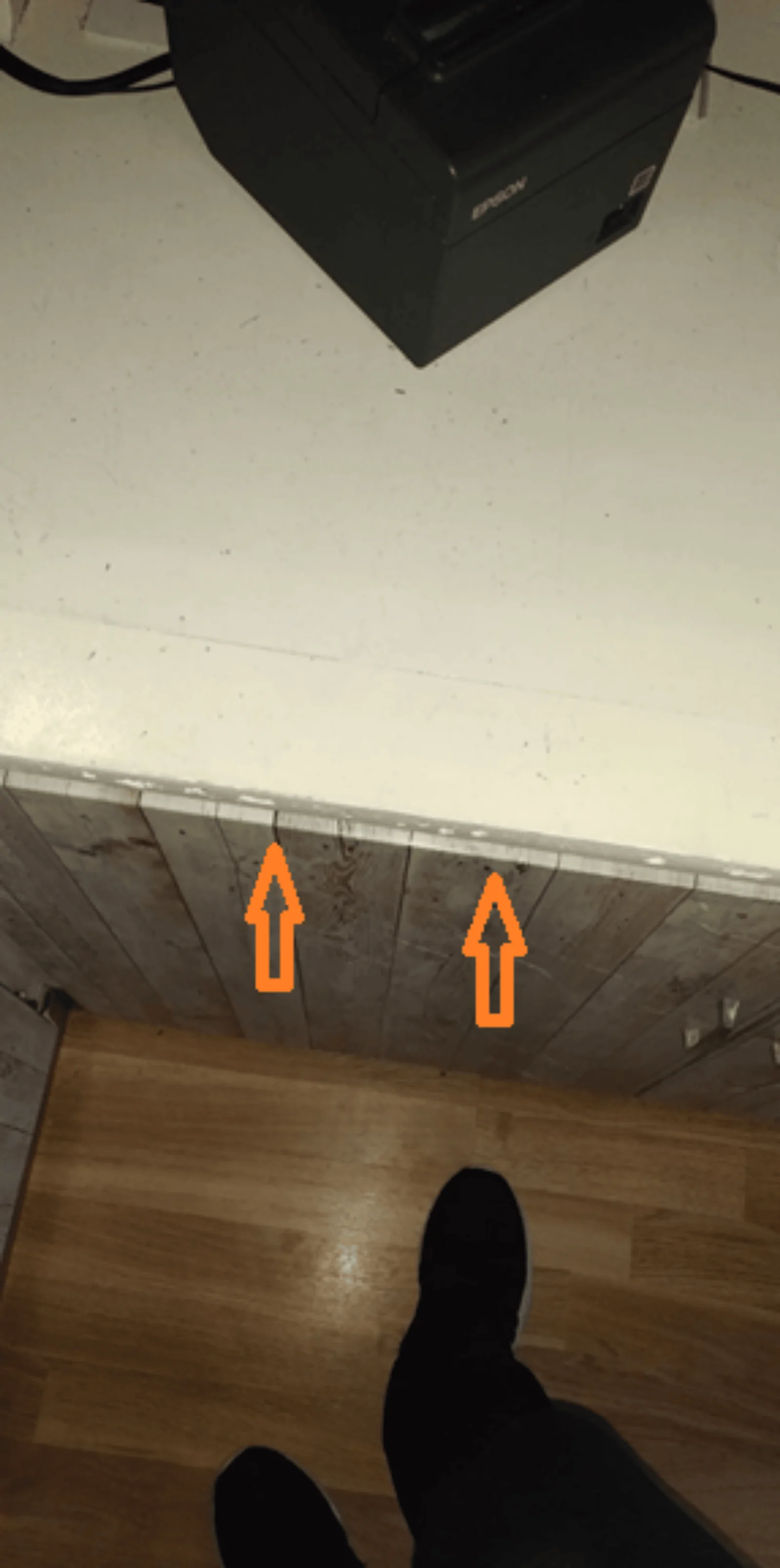
Edge of workplace table (orange arrows). The rigid horizontal edge of the furniture, positioned at the level of the injured anatomical region, indicates the potential contact surface responsible for the observed injury pattern.

On further review of her occupational history, the patient reported habitually resting the anterolateral aspects of both thighs against the edge of the payment counter for prolonged periods while working (Figure 3). This occupational exposure provided a plausible mechanism of repetitive mechanical microtrauma and further supported the diagnosis of SL.

She was discharged with advice to modify her working habits and, if possible, change the store furniture to avoid repeated pressure on the affected areas. At outpatient follow-up, the patient reported adherence to these recommendations, and clinical examination demonstrated clear improvement of the skin depressions. This favorable evolution after removal of the presumed mechanical trigger supported the association between repetitive pressure and SL and highlighted the potential reversibility of this condition.

## Discussion

Semicircular lipoatrophy (SL) is characterized by one or more semicircular, horizontal, band-like depressions resulting from localized atrophy of subcutaneous adipose tissue, typically affecting the anterolateral aspects of the thighs [6]. The overlying skin and underlying muscles remain intact [7]. The first reported occurrence of this phenomenon was described by Gschwandtner and Münzberger in 1975 [8].

Although the pathogenesis of SL is not completely understood, repetitive local mechanical pressure resulting from mild recurrent trauma (such as prolonged pressure against an object or tight clothing) is considered the most widely accepted contributing factor [9-11]. Other environmental and occupational factors have also been suggested, including workplace ergonomics, humidity, and electrostatic phenomena; however, the exact mechanism remains uncertain.

The diagnosis of SL is primarily clinical and relies on the recognition of characteristic morphological features, including unilateral or bilateral semicircular horizontal depressions caused by localized subcutaneous tissue atrophy, usually involving the anterolateral thighs. Preservation of the overlying skin and underlying muscle, absence of significant inflammatory changes, and identification of a possible repetitive mechanical trigger are important diagnostic features. Although biopsy may demonstrate localized lipoatrophy with histopathological findings compatible with traumatic panniculitis, histopathological confirmation is not routinely required when the clinical presentation is typical. Alternative diagnoses, such as morphea and localized panniculitis, are considered less likely in the absence of inflammatory skin changes, induration, pain, systemic symptoms, or deeper tissue involvement on imaging.

The number of reported SL cases has increased substantially since the 1990s, particularly in occupational settings. In 2009, a cluster of 900 cases of SL was reported among employees of a Brussels bank over an eight-year period, 84% of whom were women working with computers [12]. In this group, spontaneous resolution of the cutaneous alterations was observed during periods away from work, with recurrence after returning to the workplace [9].

A further outbreak involving 400 cases was reported in Barcelona. Following interventions addressing possible environmental factors, including avoidance of sharp-edged table surfaces, improvement of humidity conditions, and reduction of electrical discharge, complete remission of SL was observed in 90% of patients within six months [3].

Only a limited number of cases have undergone biopsy, with findings demonstrating localized lipoatrophy compatible with traumatic panniculitis [2]. Avoidance of repeated exposure to mechanical pressure has been associated with complete or partial disappearance of lesions in most reported cases [13]. These findings suggest that, although multiple factors may contribute to SL development, repetitive mechanical pressure appears to be the most consistently reported contributing factor [14].

In the present case, SL was considered the most likely diagnosis based on the typical bilateral and symmetrical distribution of the lesions, preservation of the overlying skin, ultrasonographic demonstration of localized subcutaneous tissue loss with preserved fascia and muscle, absence of laboratory findings suggestive of systemic or autoimmune disease, and identification of repetitive occupational mechanical pressure. The improvement of the lesions after modification of working habits and avoidance of the presumed mechanical trigger further supported the diagnosis.

## Conclusions

Semicircular lipoatrophy is an uncommon but increasingly recognized occupational condition characterized by localized atrophy of the subcutaneous adipose tissue, typically affecting the anterolateral aspects of the thighs. Although its pathogenesis is not completely understood, repetitive mechanical pressure appears to be the most consistently reported contributing factor.

This case highlights the importance of considering SL in patients presenting with characteristic semicircular thigh depressions and emphasizes the value of a detailed occupational history to identify repetitive workplace-related mechanical pressure. Recognizing this association may facilitate early diagnosis, prevent unnecessary investigations, and allow the implementation of simple preventive measures, such as modifying working habits or making environmental adjustments. In typical cases, diagnosis can be established clinically and supported by imaging findings when necessary, without requiring invasive procedures. Further studies are needed to better understand the pathophysiological mechanisms underlying SL and to establish more standardized approaches for diagnosis, prevention, and management.
